# Validation of a two-item short form of the perceived health competence scale

**DOI:** 10.1186/s41687-025-00963-5

**Published:** 2025-11-14

**Authors:** Devika Nair, Jacquelyn S. Pennings, Hayden B. Bosworth, Kenneth E. Freedland, Sunil Kripalani, Elisa J. Gordon, Gurjeet S. Birdee, Justin M. Bachmann

**Affiliations:** 1 Division of Nephrology, Department of Medicine, Vanderbilt University Medical Center, Nashville, TN USA; 2 Departments of Biostatistics and Orthopaedic Surgery, Center for Musculoskeletal Research, Vanderbilt University Medical Center, Nashville, TN USA; 3 Department of Medicine, Duke University Medical Center, NC Durham, USA; 4 Department of Psychiatry, Washington University School of Medicine, St. Louis, MO USA; 5 Section of Hospital Medicine, Department of Medicine and Vanderbilt Center for Health Services Research, Vanderbilt University Medical Center, Nashville, TN USA; 6 Department of Surgery, Center for Biomedical Ethics and Society, Vanderbilt University Medical Center, Nashville, TN USA; 7 Department of Physical Medicine & Rehabilitation, Vanderbilt University Medical Center, Nashville, TN USA; 8 Division of Cardiovascular Medicine, Department of Medicine, Vanderbilt University Medical Center, Nashville, TN USA; 9 Veterans Affairs Tennessee Valley Healthcare System, 2525 West End Ave, Ste 300-A, Nashville, TN 37203 USA

**Keywords:** Perceived health competence, Self-efficacy, Validation studies, Psychometrics, Health behavior

## Abstract

**Background:**

Perceived health competence is a construct encompassing individuals’ confidence in managing their health and health-related behaviors. The 8-item Perceived Health Competence Scale (PHCS-8), developed to measure this construct, has established psychometric properties including test-retest reliability and predictive validity for health behaviors and clinical outcomes. We aimed to validate the 2-item Perceived Health Competence Scale (PHCS-2), an abbreviated version of the PHCS-8, for use in clinical and research settings where respondent burden is a primary concern.

**Methodology:**

We conducted a psychometric validation study using pooled data from 482 participants across two cohort studies conducted in an integrative medicine clinic. Participants completed the PHCS-8, PROMIS Global Health Scale (yielding physical and mental health T-scores), Socially Desirable Response Set Five-Item Survey (SDRS-5), and sociodemographic information. Psychometric evaluation included internal consistency reliability, exploratory factorial validity analysis, Bland-Altman analysis of instrument agreement, convergent validity through correlation analysis, and known-groups validity across demographic and health status subgroups.

**Results:**

Study participants had a mean age of 51 years (SD = 13.2), were 81% female, and predominantly college-educated (86%). The PHCS-2 demonstrated appropriate response distributions with optimal inter-item correlation (*r* = 0.31) and factorial validity (factor loadings: 0.569, 0.522). Criterion validity was supported by strong correlation with the PHCS-8 (*r* = 0.80) and minimal systematic bias in Bland-Altman analysis (mean difference = 0.032, 95% limits: -0.792 to 0.856). Convergent validity was demonstrated through moderate correlations with PROMIS physical health (*r* = 0.463) and mental health (*r* = 0.391) T-scores. Known-groups validity was confirmed by significant differences between individuals with above-average versus below-average physical health (Cohen’s d = 0.92) and mental health (Cohen’s d = 0.62). Discriminant validity was supported by no significant correlation with social desirability (SDRS-5, *r* = 0.013, *p* > 0.05).

**Conclusions:**

The PHCS-2 represents a psychometrically sound, brief measure of perceived health competence that maintains strong concordance with the full PHCS-8 while substantially reducing respondent burden. This validated instrument provides an efficient clinical screening method to identify patients with lower perceived health competence who may benefit from targeted self-management support interventions.

## Background

Perceived health competence, defined as an individual’s confidence in managing their health and health-related behaviors, is a useful psychological construct in research and clinical practice. The Perceived Health Competence Scale (PHCS-8) [1], an 8-item questionnaire, was developed to address the measurement gap between highly generalized self-efficacy instruments [2] and behavior-specific health efficacy scales [3]. This instrument assesses domain-specific self-efficacy focused on health management capabilities, balancing both outcome expectancies (beliefs that certain actions lead to desired health outcomes) and behavioral expectancies (beliefs about one’s personal capability to successfully execute those health-related actions) [1]. The PHCS-8 has demonstrated robust psychometric properties across diverse populations [1, 4, 5] and satisfactory test-retest reliability over time [1]. Substantial evidence supports its predictive validity, with higher PHCS-8 scores consistently associated with increased engagement in health-promoting behaviors [6], better treatment adherence [7], improved health-related quality of life [8] and enhanced clinical outcomes in both general [9] and clinical [10] populations.

However, the implementation of comprehensive psychosocial measures in clinical and research settings is constrained by respondent time limitations. To address this limitation, a condensed two-item version (PHCS-2) was developed, comprising one positively-worded and one negatively-worded statement from the original scale [7, 11, 12]. The PHCS-2 has demonstrated its utility in specific contexts, particularly among cardiovascular disease patients [7, 11, 12], where it has predicted clinically meaningful outcomes including physical activity engagement [7] and cardiac rehabilitation enrollment [12]. However, the psychometric properties of the PHCS-2 have not been examined. We aimed to systematically assess the measurement properties of the PHCS-2 using cohorts from the Mind-body Self Efficacy Study [13] and the Yoga Self-Efficacy Study [14]. This validation is significant given the need for efficient instruments to identify individuals with low perceived health competence who may benefit from targeted interventions to enhance self-management capabilities.

## Methods

This study followed COnsensus-based Standards for the selection of health Measurement INstruments (COSMIN) [15] reporting guidelines (Electronic Supplementary Material). The study protocol was deemed to be exempt from further review by the Institutional Review Board of the Vanderbilt Human Subjects Research Protection Program.

For the purposes of this study, we combined two existing datasets that had been collected for the development and validation of complementary health measures. The Mind-body Self-Efficacy Study [13] collected the PHCS-8 alongside several other psychosocial measures in a cohort of mindfulness meditation practitioners recruited via convenience sampling with a snowball component from the Osher Center for Integrative Medicine at Vanderbilt University Medical Center. Similarly, the Yoga Self-Efficacy Study [14] collected the PHCS-8 in a cohort of yoga practitioners recruited through convenience and snowball sampling from the same integrative medicine clinic. The two datasets shared numerous instruments and were pooled for the purposes of this analysis. All participants in the MSES and YSES provided informed consent.

The two items for the PHCS-2 were determined by the late Kenneth A. Wallston, PhD, the original developer of the PHCS-8 [1], for use in the Vanderbilt Inpatient Cohort Study [16]. Dr. Wallston selected these items (1: ‘It is difficult for me to find effective solutions to the health problems that come my way’ (negatively worded) and 2: ‘I am able to do things for my health as well as most other people’ (positively worded)) because they succinctly capture the core dimensions of confidence in managing health-related outcomes. This item selection process, based on expert judgment, represents a theoretically-informed approach that leverages experience in health psychology and self-efficacy research rather than a data-driven item reduction process. Expert-driven item selection aligns with established practices in scale development where construct validity is prioritized, particularly when creating abbreviated versions intended for practical utility in clinical and research settings [15].

Demographic information collected from participants included age (continuous), sex (male, female), educational attainment (categorized as: 8th grade or less, some high school, high school graduate/GED, vocational/technical school, associate degree/some college, bachelor’s degree, and advanced degree), and annual household income (categorized in seven increments from less than $15,000 to $100,000 or more with an option to decline). For analytical purposes, education was recategorized into three groups: pre-bachelor’s degree, bachelor’s degree, and advanced degree. Income was similarly recategorized into four groups: <$35,000, $35,000 to <$75,000, $75,000 + and decline to answer. The Patient-Reported Outcomes Measurement Information System (PROMIS) Global Health Scale [17] was used to assess health-related quality of life. This 10-item measure yields two primary domains: Global Physical Health and Global Mental Health. Following standard PROMIS scoring procedures, raw scores were converted to T-scores with a mean of 50 and standard deviation of 10 in the reference population, with higher scores indicating better health status. Known-groups validity analyses examined whether the PHCS-2 could differentiate between participants categorized as having below-average (T-score < 50) or above-average (T-score ≥ 50) physical and mental health. To assess potential response bias, the Socially Desirable Response Set Five-Item Survey (SDRS-5) [18] was administered. This 5-item scale measures the tendency to provide socially desirable responses rather than true self-assessments. Each item is scored dichotomously (0 = no, 1 = yes), with total scores ranging from 0 to 5. Higher scores indicate greater propensity for socially desirable responding.

Per COSMIN guidelines [15], we hypothesized a priori that the 2-item Perceived Health Competence Scale (PHCS-2) would demonstrate: [1] strong concordance with the full PHCS-8, evidenced by a high correlation coefficient (*r* >0.70) and acceptable agreement on Bland-Altman analysis with minimal systematic bias; [2] adequate construct validity through moderate positive correlations (*r* ≈ 0.4–0.6) with PROMIS Global Physical Health and Global Mental Health T-scores, reflecting convergent validity with established health-related quality of life measures; and [3] robust known-groups validity, with significantly higher scores among participants with above-average physical and mental health (T-scores ≥ 50) compared to those with below-average health (T-scores < 50) as demonstrated by effect sizes (Cohen’s d) of at least 0.5.

Descriptive statistics were calculated for all demographic variables, with continuous measures presented as means and standard deviations (SD), and categorical variables presented as frequencies and percentages. For psychometric evaluation of the PHCS-2, we first conducted item-level analyses including examination of response distributions, means, and standard deviations for both PHCS-2 and PHCS-8 items. To assess structural validity, exploratory factor analysis (EFA) [19] was performed on the eight PHCS-8 items, including the two PHCS-2 items, with principal axis factoring (PAF) as the extraction method. Although initial eigenvalues analysis yielded two factors (eigenvalues of 4.2 and 1.04), visual inspection of the scree plot strongly supported a unidimensional structure. Given the theoretical underpinning of the PHCS as a unidimensional construct and the marginally exceeding second eigenvalue (1.04), a single-factor solution was specified. Factor loadings ≥ 0.5 were considered adequate for construct representation. This approach addressed the methodological artifact commonly observed with balanced scales containing both positively and negatively worded items, which can sometimes produce artificial factor separation based on item wording direction rather than conceptual distinction [20]. Reliability assessment of the PHCS-2 employed multiple approaches. Inter-item correlation was considered the primary reliability indicator for the 2-item scale, with values between 0.30 and 0.50 considered optimal per established psychometric guidelines [21, 22]. Cronbach’s alpha was also calculated for comparative purposes but was interpreted with consideration of its mathematical constraints in very brief scales, where conventional thresholds (≥ 0.70) are rarely attainable [23]. For the 8-item PHCS-8, standard reliability criteria were applied (α ≥ 0.70). To assess construct validity, we examined bivariate relationships between PHCS-2 scores and theoretically related constructs using Pearson correlation coefficients for normally distributed variables and Spearman correlation coefficients for non-normally distributed measures (e.g., SDRS-5).

Agreement between PHCS-2 and PHCS-8 was evaluated using several approaches: (1) Pearson correlation coefficient to assess linear association (2), Bland-Altman analysis [24] to examine systematic differences across the measurement range, and (3) concordance correlation coefficient to evaluate absolute agreement. To address potential inflation of correlation estimates due to item overlap, a sensitivity analysis was conducted comparing PHCS-2 with only the non-overlapping items from the full scale (PHCS-6, excluding the two PHCS-2 items). This analysis included calculation of: (a) Pearson correlation coefficients between PHCS-2 and both PHCS-8 and PHCS-6, (b) comparative coefficient of determination (R²) values to quantify explained variance, and (c) derivation of regression equations for estimating both PHCS-8 and PHCS-6 scores from PHCS-2 responses. In the primary analysis, the Bland-Altman analysis included calculation of mean differences and 95% limits of agreement, with visual inspection of plots to identify potential bias. To examine known-groups validity, PHCS-2 scores were compared across demographic and health status categories using independent samples t-tests and one-way ANOVA with post-hoc Tukey tests where appropriate. Effect sizes were calculated using Cohen’s d for two-group comparisons, with values between 0.2, 0.5, and 0.8 indicating small, medium, and large effects, respectively.

Alpha was set at 0.05 and statistical analyses were conducted using SPSS version 30.0 [25] and R version 4.4.2 [26].

A total of 3 participants were missing age, sex, education, PROMIS Global Physical Health and PROMIS Global Mental Health. We conducted a complete case analysis for statistical analyses using these variables. There were no missing data for the PHCS-8 (or PHCS-2) items, so no imputation or special handling was necessary for these analyses.

## Results

Baseline characteristics of study participants from the combined Mind-body Self-Efficacy Study (*n* = 184, 38.2%) and Yoga Self Efficacy Study (*n* = 298, 61.8%) are displayed in Table 1. The sample had a mean age of 50.8 years (SD = 13.2) and was predominantly female (80.9%). Participants were highly educated, with 86.1% having attained at least a bachelor’s degree and 58.9% holding advanced degrees. Mean PROMIS Global Physical Health T-score was 53.3 (SD = 6.1) and Global Mental Health T-score was 50.4 (SD = 7.7), both slightly above the population reference mean of 50. The mean score on the SDRS-5 scale was relatively low at 0.5 (SD = 1.1) as compared to prior studies [18, 27].

**Table 1:** Baseline characteristics of the combined Mind-body self efficacy study and yoga self efficacy study datasets (*N* = 482)

Variable	*N*
*Study*	
Mind-body Self Efficacy Study	184 (38.2%)
Yoga Self Efficacy Study	298 (61.8%)
Age, mean, SD	50.8 (13.2)
*Sex*	
Male	89 (18.5%)
Female	390 (80.9%)
*Education*	
8th grade or less	1 (0.2%)
Some high school	0 (0.0%)
High school graduate/GED	7 (1.5%)
Vocational/Technical school	9 (1.9%)
Associate degree/some college	47 (9.8%)
Bachelor’s degree	131 (27.2%)
Advanced degree	284 (58.9%)
*Income*	
Less than $15,000	26 (5.4%)
$15,000 - $24,999	27 (5.6%)
$25,000 - $34,999	32 (6.6%)
$35,000 - $49,999	45 (9.3%)
$50,000 - $74,999	78 (16.2%)
$75,000 - $99,999	73 (15.1%)
$100,000 or more	139 (28.8%)
Decline to answer	62 (13.0%)
PROMIS Global Physical Health T-score, mean, SD	53.3 (6.1)
PROMIS Global Mental Health T-score, mean, SD	50.4 (7.7)
Socially Desirable Response Set Five-Item	0.5 (1.1)

SD, standard deviation; PROMIS; Patient-Reported Outcomes Measurement Information System

A total of 3 participants were missing age, sex, education, PROMIS Global Physical Health and PROMIS Global Mental Health

Descriptive statistics for item-level responses for both the PHCS-8 and PHCS-2 instruments are displayed in Table 2. The two PHCS-2 items demonstrated appropriate response distributions across the 5-point Likert scale of each response (strongly disagree to strongly agree). Exploratory factor analysis revealed that both PHCS-2 items loaded adequately onto the primary factor extracted from the full PHCS-8, with factor loadings of 0.569 for the negatively worded item and 0.522 for the positively worded item, supporting the construct validity of the abbreviated scale. For the negatively worded item, the mean score was 2.05 (SD = 0.89), with 76.2% of respondents selecting “Strongly disagree” or “Disagree,” indicating high perceived competence in finding health solutions. The positively worded item showed a mean of 4.14 (SD = 0.84), with 84.2% of participants selecting “Agree” or “Strongly agree.” The full PHCS-8 demonstrated excellent internal consistency (Cronbach’s α = 0.872). Internal consistency reliability of the PHCS-2 as measured by Cronbach’s alpha was 0.41, as expected for a two-item scale due to mathematical constraints. The inter-item correlation of the PHCS-2 items was *r* = 0.31 (*p* < 0.001), falling within the predefined optimal range (0.30–0.50), indicating moderate internal consistency and suggesting adequate item homogeneity without redundancy.

**Table 2:** Item-level responses and descriptive statistics for the 8-item and 2-item perceived health competence scales (*N* = 482)

		%				
Item-level Response	Mean (SD)	Strongly disagree	Disagree	Neither agree nor disagree	Agree	Strongly agree
It is difficult for me to find effective solutions to the health problems that come my way (PHCS-2)*	2.05 (0.89)	27.4	48.8	16.2	6.6	1.0
I am able to do things for my health as well as most other people (PHCS-2)	4.14 (0.84)	1.2	3.5	11.1	48.1	36.1
Typically, my plans for my health don’t work out well*	1.86 (0.80)	33.6	52.3	9.6	3.7	0.8
I succeed in the projects I undertake to improve my health	3.91 (0.81)	1.0	3.9	19.1	54.4	21.6
I find my efforts to change things I don’t like about my health are ineffective*	2.10 (0.95)	25.1	52.3	12.6	7.3	2.7
No matter how hard I try, my health just doesn’t turn out the way I would like*	1.89 (0.90)	36.7	45.4	11.6	4.4	1.9
I handle myself well with respect to my health	4.06 (0.83)	0.4	6.0	11.4	51.5	30.7
I’m generally able to accomplish my goals with respect to my health	3.90 (0.86)	1.9	5.0	16.7	54.4	22.0

SD, standard deviation; PHCS-2, 2-item Perceived Health Competence Scale

The first two items (bold) comprise the PHCS-2 questions

*Denotes items that are reverse-coded

A bivariate correlation matrix between the PHCS-2 and other study measures is presented in Table 3. The PHCS-2 demonstrated a strong positive correlation with the full PHCS-8 (*r* = 0.798, *p* < 0.001), indicating substantial shared variance between the abbreviated and original scales. Further analysis revealed that both individual PHCS-2 items correlated significantly with the full PHCS-8: the negatively worded item demonstrated a strong correlation (*r* = 0.69, *p* < 0.001), as did the positively worded item (*r* = 0.599, *p* < 0.001), confirming that each component independently captures the core construct. Construct validity was supported by moderate positive correlations between the PHCS-2 and PROMIS Global Physical Health T-scores (*r* = 0.463, *p* < 0.001) and PROMIS Global Mental Health T-scores (*r* = 0.391, *p* < 0.001), aligning with theoretical expectations that higher perceived health competence would correlate with better self-reported physical and mental health status. The PHCS-2 demonstrated a negligible correlation with age (*r* = 0.122, *p* < 0.01), indicating that perceived health competence as measured by the PHCS-2 is largely independent of participant age. No significant correlation was observed between the PHCS-2 and social desirability (SDRS-5) scores (*r* = 0.013, *p* > 0.05), demonstrating discriminant validity and indicating minimal influence of response bias on PHCS-2 measurements. The pattern of correlations for the PHCS-2 was consistent with that of the PHCS-8, though the magnitude of associations between the PHCS-8 and health-related measures was somewhat stronger (*r* = 0.550 for Physical Health; *r* = 0.503 for Mental Health, both *p* < 0.001).

**Table 3:** Correlation matrix for the 2-item perceived health competence scale with other study measures

	PHCS-2	PHCS-8	PROMIS-GPH T-score	PROMIS-GMH T-score	SDRS-5
PHCS-2	--				
PHCS-8	0.798^***^	--			
PROMIS-GPH T-score	0.463^***^	0.550^***^	--		
PROMIS-GMH T-score	0.391^***^	0.503^***^	0.485^**^	--	
SDRS-5	0.013	− 0.053	− 0.040	0.042	--
Age	0.122^**^	0.139^**^	0.094^*^	0.218^***^	0.059

*p < 0.05; **p < 0.01; ***p < 0.001

PHCS-2, 2-item Perceived Health Competence Scale; PHCS-8, 8-item Perceived Health Competence Scale; PROMIS, Patient-Reported Outcomes Measurement Information System; GPH, Global Physical Health; GMH, Global Mental Health; SDRS-5, Socially Desirable Response Set Five-Item scale

Pearson’s correlation coefficients were used for all correlations except for the SDRS-5, for which Spearman’s correlation coefficients were used due to skewed distribution of this scale

**Fig. 1:**
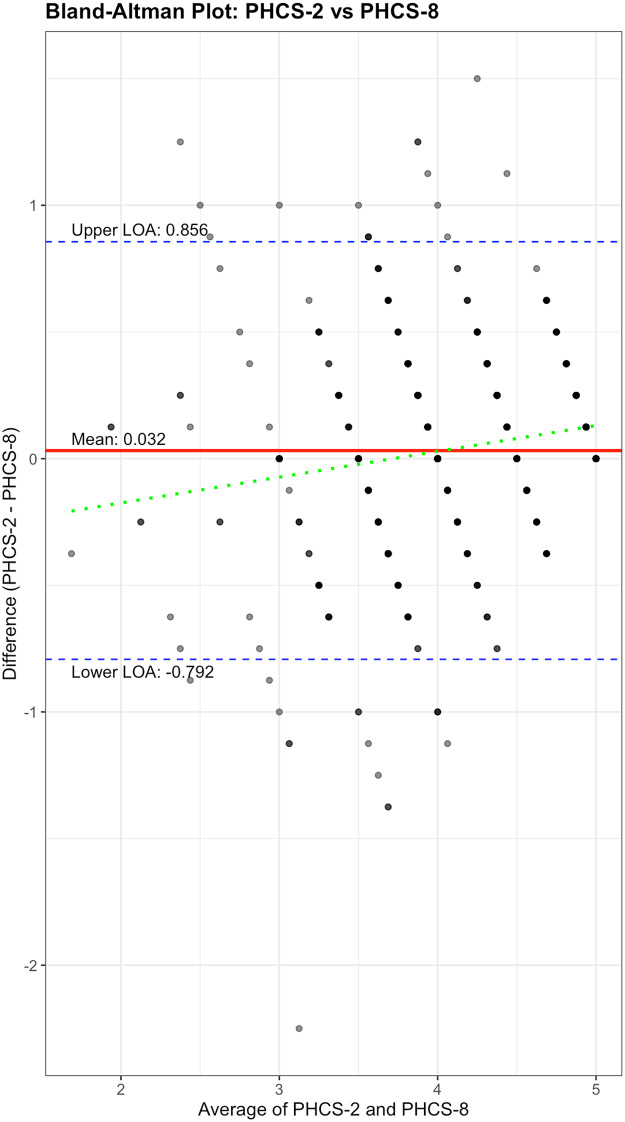
Bland-Altman Plot comparing perceived health competence scale 2-item (PHCS-2) and 8-item (PHCS-8) measurements. The y-axis shows the difference between the two measures (PHCS-2 minus PHCS-8), while the x-axis shows the mean of both measurements for each participant. The horizontal lines represent the mean difference (middle line) and 95% limits of agreement (upper and lower lines)

The Bland-Altman analysis demonstrated acceptable agreement between the PHCS-2 and PHCS-8 instruments (Figure 1). The mean difference between measures was 0.032, indicating a small systematic bias with the PHCS-2 scoring marginally higher than the PHCS-8 across the measurement range, and 95% limits of agreement ranged from − 0.792 to 0.856 with 93.6% of measurements falling within these limits. Further analysis of absolute differences revealed that 63.3% of participants had PHCS-2 and PHCS-8 scores within 0.25 points of each other, and 76.6% had scores within 0.4 points, demonstrating substantial practical concordance between the measures. Linear regression analysis yielded a significant predictive equation (PHCS-8 = 1.066 + 0.729 × PHCS-2, R² = 0.638, *p* < 0.001), which serves as a conversion formula to estimate PHCS-8 scores when only PHCS-2 data are available. Statistical analysis of proportional bias revealed a small but statistically significant slope coefficient (β = 0.102, *p* < 0.001), indicating that the magnitude of difference between measures varied slightly across the spectrum of perceived health competence, with greater discrepancies at higher competence levels. Despite this minor proportional bias, the concordance correlation coefficient between measures remained substantial at 0.79 (95% CI: 0.76 to 0.82). The relatively narrow dispersion of difference scores around the mean difference line supports the utility of the PHCS-2 as an efficient proxy measure for the full PHCS-8 scale. Additional measurement error analysis revealed a standard error of measurement (SEM) of 0.53 (13% of mean score) and smallest detectable change (SDC) of 1.46 (36.2% of mean score), providing a preliminary indication of score precision.

A sensitivity analysis comparing PHCS-2 with only the non-overlapping items from the full scale (PHCS-6, excluding the two PHCS-2 items) was conducted to address potential inflation of correlation due to item overlap. This analysis revealed a correlation of *r* = 0.657 between PHCS-2 and PHCS-6, compared to *r* = 0.798 between PHCS-2 and the full PHCS-8. The PHCS-2 still accounted for substantial variance in the non-overlapping items (R²=0.432), supporting the abbreviated scale’s ability to capture the core construct independent of item overlap. The predictive relationship between the brief and extended measures was further demonstrated by the regression equation for estimating the non-overlapping items (PHCS-6 = 1.422 + [0.638 × PHCS-2]).

Given the concentration of PHCS-2 scores above 3.0, we examined whether agreement varied across the measurement spectrum. Stratified analysis revealed consistent agreement across low (< 3.5; *n* = 137, 28.4%), moderate (3.5–4.5; *n* = 264, 54.8%), and high (> 4.5; *n* = 81, 16.8%) PHCS-2 score ranges. The low score group showed a small negative bias (mean difference=-0.25) but maintained acceptable limits of agreement (-1.25 to 0.75). Proportional bias analysis confirmed a statistically significant but clinically minimal relationship (β = 0.102, *p* < 0.001).

Table 4 presents the known-groups validity analysis for the PHCS-2, focusing on its ability to discriminate between clinically meaningful subgroups. Significant differences in PHCS-2 scores were observed between participants from different cohorts, with the Yoga Self-Efficacy Study participants reporting higher perceived health competence than Mind-body Self-Efficacy Study participants (4.12 vs. 3.93, *p* = 0.004), though the effect size was small (Cohen’s d = 0.27). No statistically significant differences were found across education or income categories (*p* = 0.457 and *p* = 0.538, respectively), and differences by sex approached but did not reach statistical significance (female: 4.07 vs. male: 3.93, *p* = 0.072). The PHCS-2 demonstrated particularly robust discriminative capacity based on health status, with substantially higher scores among participants reporting above-average physical health compared to those with below-average physical health (4.16 vs. 3.57, *p* < 0.001, Cohen’s d = 0.92). Similarly, participants with above-average mental health reported significantly higher PHCS-2 scores than those with below-average mental health (4.22 vs. 3.81, *p* < 0.001, Cohen’s d = 0.62). The large effect sizes observed for both physical and mental health comparisons provide strong evidence for the PHCS-2’s construct validity and its ability to distinguish between clinically relevant patient subgroups.

**Table 4:** PHCS-2 scores stratified by demographic characteristics and health status

Group	*N*	PHCS-2 Mean (SD)	*p*-value	Effect Size (Cohen’s d)
*Study*				
MSES	184	3.93 (0.75)	0.004	0.27
YSES	298	4.12 (0.63)		
*Sex*				
Male	89	3.93 (0.70)	0.072	0.21
Female	390	4.07 (0.68)		
*Education*				
Pre-Bachelor’s	64	3.95 (0.79)	0.457	-
Bachelor’s degree	131	4.07 (0.69)		
Advanced degree	284	4.06 (0.66)		
*Income*				
<$35k	85	3.97 (0.66)	0.523	-
$35k to <$75k	123	4.03 (0.71)		
$75K+	212	4.06 (0.70)		
Decline to answer	62	4.13 (0.62)		
*PROMIS Global Physical Health*				
Below average (< 50)	96	3.57 (0.78)	< 0.001	0.92
Above average (50+)	383	4.16 (0.61)		
*PROMIS Global Mental Health*				
Below average (< 50)	200	3.81 (0.73)	< 0.001	0.62
Above average (50+)	279	4.22 (0.60)		

MSES, Mind-body Self Efficacy Study; YSES, Yoga Self-Efficacy Study; PHCS-2, 2-item Perceived Health Competence Scale; PROMIS, Patient-Reported Outcomes Measurement Information System

To examine performance in individuals with health challenges, we conducted sensitivity analyses in participants with PROMIS Global Physical or Mental Health T-scores below 50 (*n* = 243, 50.4%). These participants represent individuals with health status below the general population average, a clinically relevant range for screening applications. The PHCS-2 maintained strong psychometric properties in this subgroup, with correlation to PHCS-8 of 0.747 (vs. 0.798 in full sample), comparable agreement (mean difference = 0.057, 95% limits of agreement: -0.863 to 0.976), and minimal floor effects (0.4%). The scale successfully discriminated between physical-only (mean = 3.80) versus mental-only (mean = 4.01) health limitations (*p* = 0.025, Cohen’s d = 0.37).

## Discussion

Our findings support the PHCS-2 as a psychometrically sound brief measure of perceived health competence. Analyses confirmed acceptable factorial validity, with both items loading adequately on the primary factor and demonstrating appropriate internal consistency without redundancy. The abbreviated scale maintained strong convergent validity with the original PHCS-8 and established meaningful relationships with theoretically-related health constructs. Sensitivity analysis further demonstrated substantial correlation between the PHCS-2 and non-overlapping items from the full scale (*r* = 0.657), providing methodologically rigorous evidence that the abbreviated measure captures the core construct beyond item redundancy. Instrument agreement analyses revealed minimal systematic bias between the two measures, with substantial concordance across the measurement spectrum. Notable discriminative properties were observed, particularly in the scale’s ability to differentiate between participants with varying physical and mental health status, evidenced by robust effect sizes. These analyses affirm the PHCS-2’s utility as an efficient alternative to the full instrument for assessing perceived health competence in research and clinical contexts.

The original validation of the PHCS-8 [1] was grounded in Social Cognitive Theory and the matching principle [28], and the PHCS-2 shares this theoretical framework. Newer theoretical developments also lend context to both the PHCS-8 and the PHCS-2. For instance, within the Health Action Process Approach [29], perceived health competence functions as measure of confidence that bridges the intention-behavior gap, a theoretical area where brief assessment is particularly valuable. Similarly, the PHCS-2 aligns with the Common-Sense Model of Self-Regulation [30] by assessing patients’ confidence in their ability to manage health threats, complementing beliefs about illness controllability. In Self-Determination Theory [31] frameworks, PHCS-2 captures perceived competence as one of the three basic psychological needs (competence, autonomy, relatedness) influencing autonomous health motivation. This theoretical versatility positions the PHCS-2 as particularly useful in health care settings where multiple health behavior changes are simultaneously targeted.

The observed psychometric properties of the PHCS-2 in this study align with and extend previous research on the association of the PHCS-2 with various health outcomes. In prior work, the PHCS-2 demonstrated a nonlinear relationship with physical activity in coronary heart disease patients [11], with the strongest effects at lower perceived health competence values and significantly greater impact among women compared to men. The PHCS-2 also predicted health-related quality of life and positive health behaviors, including medication adherence and dietary habits, in patients hospitalized with cardiovascular disease [7]. The PHCS-2 is also associated with a significantly higher odds of cardiac rehabilitation enrollment in patients hospitalized with acute coronary syndrome [12]. These findings across diverse populations and outcomes provide substantial convergent validity for the PHCS-2 as a psychometrically robust measure of perceived health competence. The current study corroborates this convergent validity through further psychometric assessment.

Of note, the observed relationships between PHCS-2 and both physical (*r* = 0.463) and mental health (*r* = 0.391) status are somewhat attenuated compared to correlations reported with the full PHCS-8 in previous studies [1, 6, 8, 10], which typically showed *r* = 0.5–0.7. This attenuation is consistent with measurement theory when using abbreviated scales [32, 33]. While previous research has primarily validated the PHCS-2 in cardiovascular cohorts [7, 11, 12], this study extends validation to individuals engaged in complementary health practices, demonstrating broader applicability while maintaining comparable psychometric characteristics. This congruence with prior findings suggests the PHCS-2 retains the fundamental properties of its parent instrument across different populations while substantially reducing response burden.

From a research perspective, the instrument’s brevity permits inclusion in large-scale epidemiological studies, pragmatic trials, and population health surveys where respondent burden represents a significant methodological constraint. These general population applications are particularly relevant given the measure’s validation in a non-clinical sample. National health surveys could efficiently incorporate the PHCS-2 to monitor population-level health confidence trends, while workplace wellness programs and community-based prevention trials could also benefit from a brief measure. The established concordance with the PHCS-8 supports the PHCS-2’s use as an efficient proxy measure in contexts where the full scale would be impractical, such as ecological momentary assessment (EMA) protocols or multi-measure batteries. The PHCS-2 is particularly appropriate for EMA since prior work established that brief measures containing fewer than five items are essential for valid EMA data collection [34]. Digital health interventions using smartphone apps similarly require ultra-brief assessments, making the two-item scale ideal for repeated measurements in real-world settings. The PHCS-2 facilitates nuanced analyses of health self-efficacy as a potential mediator or moderator of intervention effects, particularly when examining mechanisms underlying self-management outcomes.

The PHCS-2 also has considerable potential for implementation across clinical contexts where measurement efficiency is paramount. Instrument brevity is a critical factor for successful implementation of patient-reported outcomes, with measures requiring less than one minute demonstrating substantially higher adoption rates across diverse healthcare settings as compared to longer instruments [35]. In clinical settings, this abbreviated scale could serve as a rapid screening instrument to identify patients with low perceived health competence who may benefit from targeted self-management support interventions. Perceived competence functions as a key mediator of intervention success [36], suggesting that screening with the PHCS-2 could enable clinicians to differentiate between patients requiring standard care and those needing more intensive support. Several evidence-based approaches, including motivational interviewing [37], structured self-management education [38] and tailored coaching interventions [39] are particularly effective among individuals with lower baseline self-efficacy. The PHCS-2’s robust discriminative validity for physical and mental health status suggests utility for risk stratification, potentially identifying individuals at elevated risk for suboptimal treatment adherence, limited engagement in rehabilitation programs, or poorer health outcomes. This aligns with prior work demonstrating that tailoring intervention intensity based on patient activation levels yielded superior self-management outcomes compared to standardized approaches [40]. The PHCS-2 could also be incorporated into electronic health records as a patient-reported outcome measure, enabling longitudinal monitoring of patients’ perceived health management capabilities.

Future research should focus on expanding the psychometric evaluation and applied utility of the PHCS-2 across diverse contexts. Longitudinal studies examining the measure’s temporal stability (test-retest reliability) and sensitivity to change following targeted interventions would substantiate its value for monitoring clinical trajectories. Validation across heterogeneous populations (particularly underrepresented minority groups, individuals with low health literacy, and those managing multiple chronic conditions) could address potential measurement invariance concerns. Finally, investigations examining threshold values for clinically meaningful differences in PHCS-2 scores could establish interpretive guidelines for practitioners.

Our study has limitations. First, while the PHCS-8 demonstrated robust test-retest reliability across varying time intervals [1], serial PHCS-2 measurements were not available. However, the strong correlation between PHCS-2 and PHCS-8 (*r* = 0.798) suggests the abbreviated scale likely shares similar temporal stability properties with its parent instrument. Second, the sample characteristics (predominantly female and highly educated) could limit generalizability due to potential selection bias toward individuals with higher health literacy and engagement in complementary health approaches. Nevertheless, the psychometric properties observed align with previous PHCS-2 analyses in more diverse populations, particularly cardiovascular cohorts, suggesting that the instrument maintains its measurement integrity across different demographic groups. Third, despite acceptable agreement between the PHCS-2 and PHCS-8, the observed proportional bias (β = 0.102) suggests that discrepancies between measures vary across the measurement spectrum with potentially greater divergence at higher competence levels. This bias is relatively small in magnitude, however, and did not impair the PHCS-2’s ability to discriminate between clinically meaningful subgroups, as evidenced by the robust effect sizes observed for physical health (Cohen’s d = 0.92) and mental health (Cohen’s d = 0.62) comparisons. Fourth, participants completed the PHCS-2 items as part of the full PHCS-8, potentially influencing responses through context effects. Future research administering the PHCS-2 independently would provide valuable evidence about its performance as a standalone measure. Lastly, the relatively low proportion of participants scoring below 3.5 on the PHCS-2 (28%), together with the higher income and education levels of the cohort, underscores the need for additional validation in more socioeconomically disadvantaged and clinically diverse populations.

## Conclusion

Our analyses support the psychometric integrity of the PHCS-2 as an efficient alternative to the full PHCS-8 instrument, demonstrating acceptable reliability, robust construct validity, and strong discriminative capacity while substantially reducing respondent burden. The observed concordance between the abbreviated and full scales suggests the PHCS-2 offers a pragmatic measurement solution in time-constrained clinical environments and large-scale research contexts. The instrument’s ability to distinguish meaningfully between individuals with differing physical and mental health status underscores its utility for identifying patients who may benefit from targeted self-management interventions. Ultimately, the PHCS-2 offers clinicians and researchers a psychometrically sound, brief assessment of perceived health competence that balances measurement precision with practical utility in contemporary health care environments.

## Data Availability

The datasets used and analyzed during the current study are not publicly available due to data stewardship considerations concerning the original context of data collection and specific permissions granted for this secondary use. Please contact the corresponding author for data requests.

## Abbreviations

ANOVA: Analysis of Variance
COSMIN: COnsensus-based Standards for the selection of health Measurement INstruments
EFA: Exploratory Factor Analysis
EMA: Ecological Momentary Assessment
GMH: Global Mental Health
GPH: Global Physical Health
MSES: Mind-body Self-Efficacy Study
PAF: Principal Axis Factoring
PHCS-2: 2-item Perceived Health Competence
PHCS-8: 8-item Perceived Health Competence Scale
PROMIS: Patient-Reported Outcomes Measurement Information System
SDC: Smallest Detectable Change
SDRS-5: Socially Desirable Response Set Five-Item Survey
SEM: Standard Error of Measurement
YSES: Yoga Self-Efficacy Study
